# Motivational Drive in Non-copulating and Socially Monogamous Mammals

**DOI:** 10.3389/fnbeh.2019.00238

**Published:** 2019-10-04

**Authors:** Wendy Portillo, Raúl G. Paredes

**Affiliations:** ^1^Instituto de Neurobiología, Universidad Nacional Autónoma de México, Mexico City, Mexico; ^2^Escuela Nacional de Estudios Superiores, Unidad Juriquilla, Universidad Nacional Autónoma de México, Mexico City, Mexico

**Keywords:** sexual motivation, polygamy, monogamy, wanderer, non-copulating males, asexuality

## Abstract

Motivational drives guide behaviors in animals of different species, including humans. Some of these motivations, like looking for food and water, are crucial for the survival of the individual and hence for the preservation of the species. But there is at least another motivation that is also important for the survival of the species but not for the survival of the individual. Undoubtedly, sexual motivation is important for individuals to find a mate and reproduce, thus ensuring the survival of the species. In species with sexual reproduction, when males find a female in the appropriate hormonal conditions, they will display sexual behavior. However, some healthy males do not mate when they have access to a sexually receptive female, even though they are repeatedly tested. These non-copulating (NC) individuals have been reported in murine, cricetid and ungulates. In humans this sexual orientation is denominated asexuality. Asexual individuals are physically and emotionally healthy men and women without desire for sexual intercourse. Different species have developed a variety of strategies to find a mate and reproduce. Most species of mammals are polygamous; they mate with one or several partners at the same time, as occur in rats, or they can reproduce with different conspecifics throughout their life span. There are also monogamous species that only mate with one partner. One of the most studied socially monogamous species is the Prairie vole. In this species mating or cohabitation for long periods induces the formation of a long-lasting pair bond. Both males and females share the nest, show a preference for their sexual partner, display aggression to other males and females and display parental behavior towards their pups. This broad spectrum of reproductive strategies demonstrates the biological variability of sexual motivation and points out the importance of understanding the neurobiological basis of sexual motivational drives in different species.

## Introduction

Mammals display several reproductive strategies that can be influenced by population density, group size, distribution, home range size, abundance of food and resources. In mammals, the most common mating strategy is polygamy with the polygyny (one male more than one female) and polyandry (one female, more than one male, rare or inexistent in no human species) as subtypes. In polygamy, there is no sexual exclusivity and reproductive success is maximized through multiple mating partners (Kleiman, 1977). Social monogamy is a reproductive strategy in species in which resources are evenly distributed but sparse, females can disperse and have large home ranges, and males are not able to defend the access to more than one female. Also, a low density of females and food can favor monogamy. Monogamy is also present when successful rearing of offspring requires paternal and maternal care. Males help carry the litter, provide food for them and the mother when this resource is energetically costly to obtain, and the litter size is larger (Clutton-Brock and Harvey, 1978). Socially monogamous males and females after mating establish a pair bond that can last more than one reproductive cycle. However, in monogamous species some males and females do not form this pair bond and only mate opportunistically.

Interestingly, there are males and females in polygamous and socially monogamous species that do not mate even if they have the opportunity. In humans, around 1% of healthy men and women are not interested in engaging in sexual activity and are denominated as asexual. However, asexual individuals are interested in other motivational aspects of sexuality such as romantic relationships (Bogaert, 2004; Prause and Graham, 2007; Brotto and Yule, 2017; Jones et al., 2017). The biological bases of asexuality in humans are not well understood due to their complexity and ethical issues. However, the physiological bases of asexuality have been studied in murine, cricetid and ungulates, where some males do not mate even if they are tested with several sexually receptive females. In this manuscript, we will briefly outline different motivational strategies associated with reproduction in mammals and then we will describe in more detail the possible neurobiological factors associated with non-copulating (NC) males and the socially monogamous prairie vole.

In most mammals, sexual behavior consists of stereotyped movements usually organized in predictable patterns that are similar between individuals, but which vary between species. The specific patterns displayed by males and females reflect the motivational or consummatory aspects of sexual behavior. The comparative analysis between species showing different mating strategies including monogamy, polygamy and the case of asexuality could help us understand the biological variability of sexual motivational drives in mammals.

## Motivational Drive in Rodents

Under the appropriate hormonal conditions, females in estrus will display a series of stereotyped behaviors to attract a male. Originally described by Beach (1976), proceptive behaviors are displayed to attract the male and they include approach, orientation, and runaway. After a receptive female approaches the male, she positions herself placing her anogential region in contact with his face. After that, she may display hopping and darting as if running away and ear wiggling. In some rodents, these proceptive behaviors can be accompanied by scent marking and/or ultrasonic vocalizations (Gonzalez-Flores et al., 2017). After these behaviors are displayed by the female, the male will usually follow her and display mounts and intromissions. In the case of a sexually experienced male rat, he will display around 15 intromissions before ejaculating. If the female is receptive, she will arch her back, elevate the pelvis and deviate the tail. This lordosis reflex facilitates intromissions and ejaculations (Hardy and DeBold, 1972). It has been suggested that the male rat is an unconditional incentive stimulus for the female which she will approach without a previous learning or rewarding experience (for a discussion see Ågmo, 2003). Consistent with this hypothesis studies in seminatural and natural conditions have demonstrated that the female rat has a very active role in mating, controlling and spacing the stimulation she receives during a sexual interaction (McClintock and Adler, 1978). Classical studies have shown that under laboratory conditions females can also control (pace) the sexual interaction (Erskine, 1989). Many studies indicate that when subjects (males or females) pace the sexual interaction a reward state is induced that ensures that the behavior will be repeated in the future (reviewed in Paredes, 2014). Moreover, mating under pacing conditions induces the formation of new cells and neurons in the olfactory bulbs (OBs) and dentate gyrus of the hippocampus indicative of permanent plastic changes after mating (for a review see Bedos et al., 2018; Portillo et al., 2019).

Another important characteristic that is observed in natural and/or seminatural conditions is that rats are promiscuous. Usually, several females will be in estrus at the same time and they will mate with one or several partners repeatedly changing partners in the middle of copulation (McClintock and Anisko, 1982). In this way, a female could receive as first stimulation an ejaculation from a male that had been mating with another female and a male could mate with a female that has received several intromissions or ejaculations. Other studies in which subjects can choose between different mating partners indicate that the females spend more time with a male, but the preferred male is different across the estrous cycle (Ferreira-Nuño et al., 2005). It has also been shown that rats can develop conditioned mate preference for a partner that has been associated with sexual reward cues (Pfaus et al., 2001). More recent studies in seminatural observations indicate that females have a preferred male with whom they copulate more but receive intromissions and ejaculations from both the preferred and non-preferred males (Chu and Ågmo, 2014). One important characteristic of group mating is that males and females eventually receive the same amount of stimulation with both sexes controlling sexual interaction. It thus appears that sexual behavior in rats has evolved to ensure that sexual interaction will be rewarding for both sexes and hence increase the probability that the behavior will be repeated (for a discussion see Paredes, 2014).

### Non-copulating (NC) Males

Under appropriate conditions and when the female is in estrous most males will mate with her. However, it is well documented that some males will not mate even though they area repeatedly tested with receptive females. The existence of NC animals in different species confirms the biological variability in sexual motivational drives and allows the opportunity to study and understand the biological bases of asexuality (see below). NC males have been identified in sheep, guinea pigs, gerbils, hamsters, rats and mice (Whalen et al., 1961; Harding and Feder, 1976; Paredes et al., 1990; Alexander et al., 1999; Clark and Galef, 2000; Portillo et al., 2006, 2010, 2013; De Gasperín-Estrada et al., 2008; Borja and Fabre-Nys, 2012; Canseco-Alba and Rodríguez-Manzo, 2013; Mirto et al., 2017; Ventura-Aquino and Paredes, 2017). They represent between 1% and 5% of murine (Portillo et al., 2006, 2013) and 16%–20% of ungulates (Alexander et al., 1999). To our knowledge, no research group has evaluated whether there are asexual females in different mammalian species. Therefore, this is a field of great scientific potential and interest. Some studies have evaluated females that display low levels of sexual behavior. For example, Snoeren and coworkers evaluated the sexual motivation of a female rat to approach a male using an arena with two compartments. One of the compartments was empty and the other contained a sexually active male, only females were able to move from one compartment to the other. Females were classified into three groups: those that avoid the male, females that approach the male and a middle group. The females that avoid the males show low preceptive behaviors. The authors suggest that the females that avoid the males represent an animal model to evaluate hypoactive sexual desire disorder (Snoeren et al., 2011). In the following section, we will describe studies of NC males in murine and ungulates, which are the most studied species.

Several studies have suggested that NC males can have alterations in brain regions that control sexual behavior. In mammal’s the medial preoptic area (MPOA) regulates different motivated behaviors such as aggression, parental and sexual behavior (Pfaff and Baum, 2018; Yoshihara et al., 2018; Tsuneoka, 2019). With respect to sexual behavior, the MPOA modulates the appetitive (motivational) and consummatory (execution, mount, intromission and ejaculation) aspects of male sexual behavior (Paredes, 2003; Pfaff and Baum, 2018). Bilateral lesions of the MPOA eliminate consummatory components of sexual behavior in several species including fish, lizard, snake, quail, rat, guinea pig, marmoset, chicken, frog, mouse, hamster, ferret, goat, cat, dog and rhesus monkeys. On the other hand, stimulation of the MPOA induces penile erections in squirrel monkeys. In rats stimulation of this brain region increases mating; review in Paredes (2003) and references therein. The lack of sexual behavior in NC males is not associated with a decrease in plasmatic testosterone levels or a reduction of testis and seminal vesicle weight (Stefanick and Davidson, 1987). Also, males with lesions in the MPOA do not present alterations in penile erection or seminal emission (Larsson and Heimer, 1964; Lisk, 1968; Stefanick and Davidson, 1987; Liu et al., 1997). As already mentioned, the MPOA also plays a fundamental role in the appetitive components of male sexual behavior. Male rats with MPOA lesions show a decrease in the time they pursue the female. Partner preference tests have also demonstrated the importance of the MPOA in the motivational components of male sexual behavior. When given the choice to interact with a sexually receptive female or a male, both male rats and ferrets show a clear preference for the sexually receptive female. However, after bilateral lesions of the MPOA the males do not mate with the females and they show a preference for the male in both ferrets (Cherry and Baum, 1990) and rats (Paredes et al., 1998). Male rats also show a clear preference for odors from estrous females as opposed to odors from anestrous females or clean odors. Again, rats with MPOA lesions lose this preference and equally prefer estrus and anestrus female odors. This change in olfactory preference was not associated with alterations in the neuronal processing of sexually relevant odors in the accessory olfactory system (Hurtazo and Paredes, 2005).

**Figure 1:**
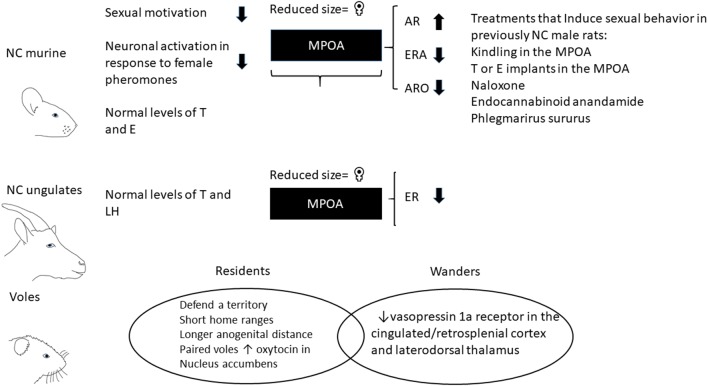
Characteristics of non-copulating males (NC) and voles with resident and wandering mating strategies. NC rats have normal plasmatic levels of testosterone (T) and estradiol (E) and ungulates have normal plasmatic levels of T and luteinizing hormone (LH). The medial preoptic area (MPOA) is involved in sexual motivation and shows a reduced size in NC rats and rams in comparison to copulating males. The MPOA in NC males has increase androgen receptors (ARs), reduce estrogen receptor alpha (ERA) and low activity of aromatase enzyme (ARO). Treatments that induce sexual activity in previously NC male rats: MPOA kindling stimulation; T or E implants in the MPOA; systemic administration of naloxone, endocannabinoid anandamide and *Phlegmarirus sururus* extract. Prairie voles are socially monogamous species. However, around 30% of the males show a promiscuous mating strategy (wanderers) which have shorter anogenital distances, decrease in oxytocin receptors in the nucleus accumbens (NAc) and vasopressin 1a receptors in the cingulated/retrospenial cortex and thalamus in comparison to males that form pair bonds (residents).

Much like males with MPOA lesions, NC male rats, do not have genital dysfunction as they show penile reflexes and spontaneous seminal emission similar to copulating males (Stefanick and Davidson, 1987). Also, NC rats and mice do not have alterations in plasmatic testosterone or estradiol levels that could explain the lack of sexual interest and systemic hormone replacement fails to induce sexual activity (Whalen et al., 1961; Stefanick and Davidson, 1987; Portillo and Paredes, 2003; Portillo et al., 2006, 2013). Although there are no differences in their plasmatic hormonal levels, NC rats have alterations in their steroid receptors. Androgen receptors (ARs) are higher and estrogen receptors alpha are lower in the MPOA of NC males and the activity of the aromatase enzyme (enzyme that converts testosterone to estradiol) is reduced in the MPOA of NC males (Portillo et al., 2006, 2007). Interestingly, our research group has demonstrated, that testosterone or estradiol implants in the MPOA induces mating in previously NC male rats (Figure 1). These effects are specific to the MPOA since estradiol or testosterone implants outside this area fail to induce sexual behavior (Antonio-Cabrera and Paredes, 2014). Similarly, NC or sexually sluggish rams do not have alterations in testosterone or luteinizing plasmatic levels. However, when copulating rams cohabit with sexually receptive females their plasmatic levels of luteinizing hormone (LH) increase. This physiological response is not observed in NC rams or in males that do not mount receptive females but display the behavior with other males (male-oriented males; Alexander et al., 1999). NC rams also have alterations in their hormone receptors. NC rams have a reduced number of estrogen receptors in the MPOA and higher number in the anterior adenohypophyses in comparison to sexually active males (Alexander et al., 1993). Moreover, studies in rats and rams have shown that the MPOA of NC or sexually sluggish males, those that do not mate consistently or take a long time to ejaculate, is smaller than that of copulating males and similar to the MPOA of females (Rhees et al., 1999; Alexander et al., 2001) suggesting that these males show neuroanatomical feminization of the MPOA.

**Figure 2:**
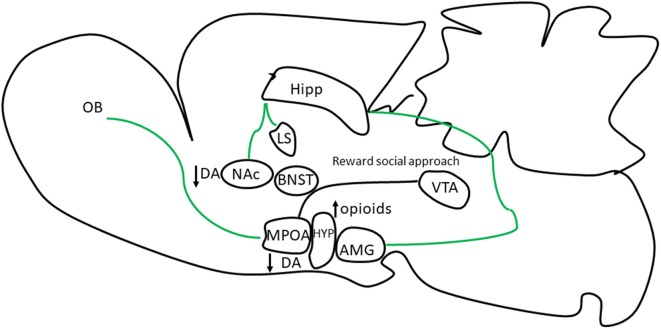
Neuronal brain regions and neuromodulators involved in sexual motivation in NC rats. Copulating male rats and mice show an increase in neuronal activity in response to odors from sexually receptive females in the olfactory bulbs (OBs) a region involved in conspecific recognition and in neuronal areas involved in the social behavior network (SBN; bed nucleus of the stria terminalis, BNST, amygdala, AMG) and MPOA, and mesolimbic reward system (MRS; NAc and ventral tegmental, VTA). However, in NC male rats and mice no increase in neuronal activity is observed in some of these neuronal regions. In NC male rats DA is not increase when males are exposed to estrous females indicative of a lack of interest reducing approach behavior to the incentive. The MPOA is an important interface with the VTA to establish a reward state that assures that the behavior will be repeated (McHenry et al., 2017).

NC male rats also have alterations in different aspects of sexual motivation. NC rats show less social behavior such as autogenital grooming and display reduced grooming partner and vocalizations than copulating males (Pottier and Baran, 1973). Our research group has shown that NC males show a reduced preference for odors or for the presence of sexually receptive females. Whereas copulating male rats and mice show a clear preference for a receptive female with whom they can mate (sexual preference) or for one they can only see, hear and smell (sexual incentive motivation) as opposed to a male or a non-receptive female, NC mice and rats do not show any preference (Portillo and Paredes, 2003, 2004; Portillo et al., 2013). In rodents, the sense of smell is very important to identify conspecifics and their pheromones. Copulating males show a strong preference for bedding exposed to secretions of receptive females as opposed to anestrous, male or clean bedding. Although NC males also choose the estrous odors this preference is significantly reduced compared to copulating males (Portillo and Paredes, 2003, 2004; Portillo et al., 2013). NC mice can discriminate volatile urine odors from males and females, but they spend less time smelling them compared with copulating males (Portillo et al., 2013). Taken together, these results suggest that NC males are not sexually motivated by the receptive females or their odors (Figure 2).

The lower preference for estrous female odors in NC males may be due to deficits in the neuronal processing of sexually relevant odors. For example, when copulating males detect odors from estrus females, the MPOA, and other neuronal regions in the vomeronasal projection pathway increase their neuronal activity, evaluated by the expression of the protein of the early gen c-Fos. On the contrary, the MPOA and central structures of the vomeronasal projection pathway in NC males do not increase their neuronal activity (Portillo and Paredes, 2004; Portillo et al., 2013). Thus, NC males have an alteration in the neuronal processing of sexually relevant cues. This reduction in neural activity could simply reflect the reduce motivation that these males have for sexually receptive females or their odors (Figure 2).

Sexual behavior can be induced in NC males using different experimental strategies. Systemic injection of the opioid receptor antagonist naloxone can induce mating behavior in formerly NC rats (Gessa et al., 1979; Canseco-Alba and Rodríguez-Manzo, 2019). Administration of the endocannabinoid anandamide induces sexual activity in 50% of previously NC male rats (Canseco-Alba and Rodríguez-Manzo, 2013). These males were able to mate 14 days after the drug treatment without needing another administration of the compound. Endocannabinoid anandamide induces sexual behavior in previously NC male rats through the activation of the CB1 cannabinoid receptor (Canseco-Alba and Rodríguez-Manzo, 2019). Endocannabinoids modulate presynaptic neurotransmitter release. Rodriguez-Manzo group reports a high proportion of NC male rats in their experiments, around 20% of their male Wistar rats were classified as NC. In our studies, using the same rat strain, we found that only around 1%–3% of the males can be classified as NC. The high frequency of NC males reported in other research groups could be due to different housing and or breeding conditions.

Another compound that induces sexual behavior in previously NC male rats is the aphrodisiac *Phlegmarirus saururus*. This compound is rich in alkaloids, principally sauroine, sauroxine and 6-hydroxylycopodine and when administered to NC males induces sexual behavior (Birri et al., 2017).

Kindling is a model of epilepsy in which an initially subconvulsive electrical stimulation of a specific region of the brain eventually develops a generalized seizure. Kindling can induce several plastic changes in the brain such as modulation of neurotransmitters (GABA), monoamines, several opioid peptides, long term potentiation (LTP) and changes in cellular protein synthesis (Gorter et al., 2016). Even though kindling did not modify sexual behavior in copulating rats when induced in the MPOA, the development of MPOA kindling in previously NC male rats induced sexual activity in seven out of nine animals. The sexual behavior displayed by previously NC male rats with MPOA kindling was very similar to that observed in copulating males. This effect of kindling over sexual behavior was specific to the stimulated area, because kindling in the AMG in NC males did not induce sexual behavior (Paredes et al., 1990). The induction of sexual activity in previously NC male rats with MPOA kindling is long lasting since males displayed sexual behavior even 8 months after kindling stimulation had ceased (Portillo et al., 2003). The induction of sexual activity in previously NC rats could be associated with changes in neuromodulatory systems, protein synthesis or LTP.

NC males are poorly studied in other species. In male Mongolian gerbils (which are socially monogamous; Scheibler et al., 2004), hormone exposure during fetal development modifies their sexual behavior when adults. Males that develop between two females have lower levels of circulating testosterone and deficits in the development of genital musculature in comparison to males gestated between two males. Around 22% of males located between two females when reach adulthood did not mount the females and when they cohabited with them, they failed to induce pregnancy. These NC gerbils show high levels of alloparental behavior, they spend 30%–50% more time caring for pups than males that developed between two males (Clark et al., 1992; Clark and Galef, 2000). Clark and Galef propose that although NC gerbils are unable to have descendants, they can increase their fitness by contributing to rear collateral kin.

From the above-described studies, it is evident that asexuality or the lack of copulation in different species has an important biological component that can modify the structure of the central nervous system and consequently its function reducing sexual motivation. The MPOA is a brain region where these changes might occur as part of the circuits controlling sexual behavior. NC males are a valuable animal model to study the factors that modulate motivational sex drive and hence sexual behavior.

## Asexuality

The NC males that have been identified in several species could be equivalent to asexual individuals in humans. However, it is necessary to recognize the limitations of these comparisons since the psychological (fantasies) and romantic aspects of human sexuality cannot be studied in animals. In general, asexual individuals are healthy men and women without physical or emotional disorders, who report low or absent sexual desire and/or attraction (erotic and sensual allure). That is, they do not feel sexual attraction to any congener (Bogaert, 2004; Prause and Graham, 2007; Brotto et al., 2010). Asexual individuals have more negative explicit and implicit attitudes toward sex as well as explicit negative attitudes toward romance (feeling of infatuation or emotional attachment) than individuals who engage in sex. Thus, asexual people have a neutral or negative view of sex, low passion but can have romantic attraction (Bogaert, 2012; Bulmer and Izuma, 2018; Zheng and Su, 2018). However, this sexual orientation does not prevent them from engaging in emotional relationships, and some of them have relationships with other asexual individuals (Bogaert, 2004; Prause and Graham, 2007; Brotto et al., 2010; Brotto and Yule, 2017; Jones et al., 2017).

Bogaert in 2004 reported that approximately 1% of the population of Britain and the United States identify themselves as asexual (Bogaert, 2004, 2015). In New Zealand students, asexual individuals represent about 2% of the population (Lucassen et al., 2011), and in Finland 3.3% and 1.5% of women and men, respectively (Höglund et al., 2014). Asexual individuals are more likely to be women (70%; Bogaert, 2012, 2015). Bogaert reported that asexual individuals, in general, experience their first sexual interaction at an older age than sexual persons and throughout their lives they have fewer sexual partners. Asexual and sexual women differ in parameters such as age, socioeconomic status, education, race, weight, age of menarche and religiosity (Bogaert, 2004). In contrast, asexual and sexual men differ in socio-economic status, education, weight and religiosity; review in Prause and Graham (2007). However, recent studies did not find significant differences between sexual and asexual individuals regarding education level and physical health (Greaves et al., 2017; Yule et al., 2017; Zheng and Su, 2018). Asexual people report more frequent anxiety disorders such as somatization, depression, more interpersonal problems and suicidal and psychotic symptoms than sexual participants (Yule et al., 2013).

Both asexual men and women report falling curiosity about sexual relationships during adolescence, but they report having less frequent sexual intercourse experience because it is unpleasant. In fact, a low percentage of asexual individuals reported to be in a relationship. Moreover, some asexual individuals who are married engage in sexual activity only to please their partners. That is, they have unwanted but consensual sex (Carrigan, 2011; Van Houdenhove et al., 2014, 2015a; Zheng and Su, 2018). Asexuality is not due to physical alterations, because asexual men do not have erection deficiencies and sometimes masturbation is pleasurable, but not sexual contact with a partner (Brotto et al., 2010). An early study found no significant differences in masturbation frequency between asexual and sexual men. However, while sexual men masturbate for reasons associated with sexual needs; their partners are not interested in sex, are unavailable, or they simply want sexual satisfaction, asexual men masturbate because they report to be bored, or because it helps them relax and/or fall sleep (Bogaert, 2012).

Asexual as well as sexual women participants respond to audiovisual erotic stimuli with an increase in genital congestion, which is an indication that they experience normal levels of genital arousal. Even though masturbation is usually enjoyable, asexual women masturbate less frequently than sexual women (Brotto and Yule, 2011, 2017; Zheng and Su, 2018). Similar to asexual men, asexual women masturbate to relax and release stress or tension and they feel that this activity is not sexual because it does not involve sexual thoughts or sexual emotions (Van Houdenhove et al., 2015a). In a recent study (Yule et al., 2017), asexual women and men reported to be less likely to masturbate for sexual pleasure or fun. Around 40% of asexual individuals reported that they had never had a sexual fantasy in comparison with sexual participants of both genders (8%). Sexual fantasies are less exciting in asexual than in sexual participants. Asexual people (12% men and 14% women) that have sexual fantasies, do not see themselves in the fantasies. Their fantasies are about other people, voyeurism and fictional human characters. Asexual men or woman reported to have fantasies that do not include sexual or romantic content, for example cuddling (Yule et al., 2017).

Although asexual individuals are not interested in the physical part of a relationship, they experience the need and desire to develop emotional bonds, and they look for the romantic side of relationships and a stable emotional partner. Some asexual men and women reported that they like kissing and cuddling but without a sexual connotation (Scherrer, 2008). Asexual individuals can self-categorize into aromantic with no romantic feelings and romantic. The ideal relationship of aromantic asexual men and women is a friendship-like interaction. On the other hand, romantic asexual people which represent the majority (79%–72%) have the same romantic desires and needs as sexual individuals. Romantic asexual men and women can be homo-romantics (14%), hetero-romantics (32%) and bi-romantics (26%). Other asexual individuals identify themselves as gender-neutral (not referring to either sex), genderqueer (individuals who see their gender as fluid or hybrid), or reject the binary between male and females (Scherrer, 2008; Brotto et al., 2010; MacNeela and Murphy, 2015; Van Houdenhove et al., 2015b; Zheng and Su, 2018).

Asexual people describe different benefits to their orientation. Among those are that they keep away from the common problems of intimate relationships, which include high risk of acquiring a sexually transmitted infection, unwanted pregnancies and finding a partner. Among the main disadvantages of asexuality are that asexual men and women are seen as less human than people with other sexual orientations, difficulties in establishing intimate non-sexual relationships and the positive effects of sex are missing. Some asexual individuals worry that there is something wrong with them and wonder if they are the only ones with this sexual orientation (MacInnis and Hodson, 2012). This could increase because there is a lack of awareness and disbelief that asexuality exists in the general population. Thus, the asexual community lacks visibility and credibility in social media and communications (MacNeela and Murphy, 2015; Robbins et al., 2016). In an attempt to reduce the lack of awareness and increase visibility asexual societies have been created. The Asexual Visibility and Education Network (AVEN) founded in 2005 stands out among them. AVEN is a social network that focuses on informing about asexuality. This network links members with scientific studies related to this orientation and makes information available to contact other asexual members with the possibility of finding an emotional partner. There is a clear need to understand the biological bases of asexuality. Due to ethical limitations, studies in humans have mainly concentrated on questionnaires and clinical descriptions. However, studies in NC animals suggest that they are present in different species representing a biological variability in which sexual motivation is reduced. More research is needed in this area, not to cure asexuality, but to understand and give support to those that could need it.

### Monogamous Prairie Vole

As already described, there are different reproductive strategies in mammals that are influenced by external and internal factors in trying to assure the survival of the species. While in mammals the most common mating strategy is polygamy, there are species that have developed a socially monogamous reproductive strategy (around 3%–9% of mammals) demonstrating the biological variability in sexual motivation. *Microtus ochrogaster* is a socially monogamous species (Lukas and Clutton-Brock, 2013). Sexually naïve females and males form long-lasting pair bonds after mating or cohabitation for at least 6 h, sharing a nest and home range, showing a preference for their sexual partner, displaying selective aggression to other males and females, defending a territory and displaying parental behavior to their pups. When the sexual partner dies, the survivor usually does not form a new pair (Getz and McGuire, 1993; Gobrogge, 2014; Walum and Young, 2018).

However, not all voles pair bond (residents), in natural and laboratory conditions some males have home ranges that overlap with territories of other males and females. These voles mate when they find an available receptive female but do not form a pair bond or defend the territory (Getz and McGuire, 1993; Carter et al., 1995; Getz and Carter, 1996; Ophir et al., 2008). These males represent around 30% of the population and have been denominated as wanderers. Females can also be wanderers but less than 15% have been found to adopt this reproductive strategy (Ophir et al., 2008). This behavioral pattern is not fixed since some wanderers had been residents or become residents during the same season. Studies have evaluated the socially monogamous or wandering reproductive strategies in voles. Resident male voles defend their territory and have shorter home ranges than wanderers (Solomon and Jacquot, 2002). Residents have more possibility to sire a litter probably by mate guardian. Resident males with litters had fewer home range overlaps than reproductively successful wanderers. As expected wandering males that sired a litter had a higher home range overlap than that of unsuccessful wanderers (Ophir et al., 2008, 2012).

In semi-natural conditions, resident male voles have longer anogenital distances than wanderers (Ophir and Delbarco-Trillo, 2007). Studies in rodents indicate that the anogenital distances depend on prenatal levels of testosterone; pre and neonatal treatment with an AR blocker (flutamide) decrease anogenital distance in male rats and impairs sexual behavior (Domínguez-Salazar et al., 2002). Male gerbils with longer anogenital distances have higher testosterone levels, higher testes weight, scent mark more frequently and display sexual behavior more than males with shorter anogenital distance (Clark et al., 1990). These results suggest that changes in testosterone levels could be associated with resident and wanderer mating strategies.

Female voles show a clear sexual preference for males with longer anogenital distances and larger testes. Male voles with longer anogenital distances had higher levels of seminal fluid and sperm than males with short anogenital distances (Ophir and Delbarco-Trillo, 2007). Thus, resident pair voles are more masculinized and fertile than wanderers. Females can identify these characteristic to choose a mate and eventually form a pair bond (Ophir and Delbarco-Trillo, 2007). These reproductive strategies have no impact on the general health of the voles since there are no significant differences in their body mass and survival (Solomon and Jacquot, 2002). Differences in neurotransmitters have been reported between residents and wanderers. In male prairie voles, vasopressin facilitates pair bonding. Moreover, vasopressin receptor 1a (V1aR) is higher in the ventral pallidum (VP) of prairie voles in comparison to polygamous *Microtus montanus* (montane voles) and *Microtus pennsylvanicus* (meadow vole; Nair and Young, 2006). Resident male voles that have extra-pair copulation (sexual infidelity) and wandering males in a seminatural enclosure show low levels of vasopressin 1a receptor V1aR expression in neuronal regions involved in spatial memory such as the posterior cingulate/retrosplenial cortex and laterodorsal thalamus. However sexual fidelity is not associated with vasopressin 1a receptor in the VP or lateral septum (LS) areas involved in pair bonding formation (Ophir et al., 2008).

Another neurotransmitter involved in pair-bonding is oxytocin. Prairie voles have a higher density of oxytocin receptors in the nucleus accumbens (NAc) medial prefrontal cortex (mPFC) and AMG compared to the closely related non-socially monogamous montane and meadow voles (Insel and Shapiro, 1992). Interestingly, the density of oxytocin receptors in the NAc and caudate putamen is highly variable in prairie voles (Ophir et al., 2012). Ophir and coworkers showed that sexual exclusivity is not related to oxytocin receptor density. They demonstrated that males that sired offspring only with their sexual partners did not differ in oxytocin receptors in the forebrain in comparison with males that sired offspring with a female that was not their partner. However, paired male voles had more oxytocin receptors in the NAc than wandering males (Ophir et al., 2012).

As already described, pair-bonding can be induced by mating or cohabitation for 6 h (Williams et al., 1992; Carter et al., 1995; Wang et al., 1997). We evaluated if mating and pair-bonding endure because they induce a positive affective state. In male voles, the pair bond resulting from mating until one ejaculation or copulation for 6 h induces a positive affective state evaluated by the conditioned place preference (CPP) test. This positive state is not induced if males are exposed to auditory, olfactory and visual stimulation with a receptive female, but without physical contact for 6 h. This rewarding state induced by mating is opioid-dependent because the administration of the opioid antagonist naloxone to males that ejaculate once or mate for 6 h blocked the induction of a reward state (Ulloa et al., 2018). Female voles that were exposed to a sexually active male without mating or that mated for 6 h or mated until one ejaculation did not develop a reward state. The failure to develop CPP and hence a reward state in female voles after mating could be due to the fact that females were not allowed to control, pace, the sexual interaction. As described above in order for sexual behavior to be rewarding in female rats, they need to pace the sexual interaction (Martinez and Paredes, 2001; Arzate et al., 2011). When females receive at least 10 intromission, the sexual stimulation is rewarding. Similarly, sexual behavior is rewarding only in those males that mate pacing the sexual interaction (Ågmo and Berenfeld, 1990; Paredes and Alonso, 1997; Martinez and Paredes, 2001; Parada et al., 2010; Pfaus et al., 2012). Further studies in female voles are needed to determine if sexual stimulation in pacing conditions induces a reward state.

A recent study demonstrated that female voles that mate, but not those exposed to a peer formed a place preference for cues associated with their mates (Goodwin et al., 2019). The differences between our study and that of Goodwin et al. (2019) is that we allowed the females to mate with the male for 6 h in each of the three reinforcing conditioning days. After mating females were returned to their home cage without the male partner. In Goodwin’s study females cohabited with the male for 12 h in the reinforcing conditioning days. Our females were sexually naïve and in order to avoid pregnancy females were ovariectomized and treated with intraperitoneal (i.p.) administration of estradiol benzoate. In the study of Goodwin et al. (2019), females were sexually experienced and had previously produced litters. Futures studies need to address possible rewarding differences between residents and wanderers.

### Neural Control of Sexual Motivation

In a recent review, we described in detail the possible neural circuits that control sexual motivation (Ventura-Aquino et al., 2018). Briefly, there are two brain circuits that have homologies in different vertebrate lineages which integrates internal and external stimuli. Both are part of the social decision-making network facilitating adaptation and survival of the individual. The first circuit is the social behavior network (SBN) important for the control of sexual behavior that includes brain regions such as the MPOA, the AMG, the anterior hypothalamus and the ventro medial hypothalamus. The second network, the mesolimbic reward system (MRS), includes the ventral tegmental area (VTA) and the NAc and is important for reward, including the reward associated with sexual incentives. When a potential mate is present and mating occurs, under appropriate conditions (pacing for the female, for example) a reward state will be induced that will favor the repetition of the behavior (Paredes, 2014).

**Figure 3:**
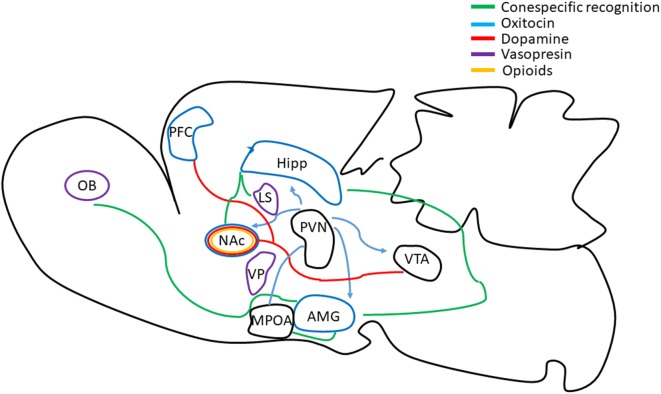
Schematic representation of emotional, reward and sensory brain circuits involved in pair-bonding formation. Recognition and memory formation of the sexual partner cues are encoded by the OB, the AMG, the MPOA, the Hipp, the NAc and the lateral septum (LS; green lines). The prefrontal cortex (PFC) and NAc modulate affiliative behavior. The VTA is known to modulate motivational, reward and emotional salient stimuli. The VP is related to the hedonic or motivational stimuli of the partner, and the paraventricular nucleus is involved in social recognition and bond separation. Oxytocin (blue lines), dopamine (red lines) and vasopressin (purple lines) play a fundamental role in pair bonding. Oxytocin is involved in individual discrimination and partner preference. Dopamine induces approach behavior facilitating partner preference without mating and is involved in pair bond maintenance. Vasopressin is relevant in social recognition, territory marking and aggressive behavior. Opioids (yellow lines) are involved in sexual and partner associated reward that contributes to the establishment of long term pair bond (review in Lieberwirth and Wang, 2016; Walum and Young, 2018).

As described above the MPOA is a key brain area regulating sexual motivation (for a review see Paredes, 2003) and the release of opioids in this brain region is important for sex to be rewarding in both males and females (Paredes, 2014). The NAc is also important for sexual motivation and dopamine (DA) is released in anticipation and prediction of reward (Berridge et al., 2009; Berridge and Robinson, 2016). Different lines of evidence indicate that a variety of events enhance DA release in the NAc, including eating, drinking, as well as aversive stimuli such as tail pinch, restraint stress, foot-shock, social defeat, and aggressive encounters (for a review see Paredes and Ågmo, 2004). Taken together these results suggest that DA is involved in the wanting response for different motivated behaviors. In rats, DA participates in the consummatory aspects of mating, whereas opioids are involved in the reward state associated with mating. In voles, mating induces oxytocin and DA release facilitating the association of sexually relevant cues of the partner with mating inducing pair bonding (Lieberwirth and Wang, 2016; Walum and Young, 2018; Figure 3).

## Conclusion

The motivational drives that control and influence sexual behavior produce great biological variability between species that induce different behavioral patterns. These behavioral patterns under the appropriate conditions allow males and females to reproduce and ensure the survival of the species. The promiscuous, monogamous and NC (asexual in humans) patterns represent different motivational drives that need to be studied to understand the neurobiology of sexual behavior.

## Author Contributions

WP and RP contributed equally to this manuscript.

## Conflict of Interest

The authors declare that the research was conducted in the absence of any commercial or financial relationships that could be construed as a potential conflict of interest.
